# Surgical Treatment for Chronic Pancreatitis: Past, Present, and Future

**DOI:** 10.1155/2017/8418372

**Published:** 2017-07-27

**Authors:** Stephanie Plagemann, Maria Welte, Jakob R. Izbicki, Kai Bachmann

**Affiliations:** Department of General, Visceral and Thoracic Surgery, University Medical Center Hamburg-Eppendorf, Martinistraße 52, 20246 Hamburg, Germany

## Abstract

The pancreas was one of the last explored organs in the human body. The first surgical experiences were made before fully understanding the function of the gland. Surgical procedures remained less successful until the discovery of insulin, blood groups, and finally the possibility of blood donation. Throughout the centuries, the surgical approach went from radical resections to minimal resections or only drainage of the gland in comparison to an adequate resection combined with drainage procedures. Today, the well-known and standardized procedures are considered as safe due to the high experience of operating surgeons, the centering of pancreatic surgery in specialized centers, and optimized perioperative treatment. Although surgical procedures have become safer and more efficient than ever, the overall perioperative morbidity after pancreatic surgery remains high and management of postoperative complications stagnates. Current research focuses on the prevention of complications, optimizing the patient's general condition preoperatively and finding the appropriate timing for surgical treatment.

## 1. Evolution of the Surgical Steps in Pancreatic Surgery

The pancreas is one of the last organs surgeons have approached in history due to its unfavorable position in the abdomen. In 1882, Karl Gussenbauer was the first one to document the treatment of a pancreatic cyst by marsupialisation. In the same year, Trendelenburg performed the first distal pancreatectomy [1]. Between 1898 and 1940, Codivilla (1898), Kausch (1909), Hirschel (1913), Tenani (1918), and Whipple (1934–1940) undertook resections of the pancreas in patients with cancer either of the ampulla of Vater or the pancreatic head. The resections in this period concentrated more on a limited resection such as enucleation.

After the first surgical approaches in resecting parts of the pancreas together with the duodenum and pylorus, surgeons started experimental approaches in dogs, trying out different possibilities in successfully accomplishing the anastomosis of the pancreas and the small intestines.

Because of the short survival time, further surgical approaches were held back in patients with pancreatic disease. Bleeding tendency was one of the major problems during that time. The following years were filled with further research on the function of the gland combined with blood chemistry.

Already in 1935, a paper was published considering early surgery for acute pancreatitis and chronic pancreatitis. The authors proposed a draining procedure by temporary drainage as an external fistula or anastomosis between the pancreas and the stomach or duodenum. In patients with a tumor in the pancreas that was suspected to be malignant, there were already the same concepts leading to resection as there are today: (1) establish the diagnosis, (2) to treat jaundice and duodenal obstruction, and (3) removing the tumor [2].

Uncontrollable hemorrhagic complications were a feared complication when proceeding with resections of the head of the pancreas due to the amount of large blood vessels nearby. The detection of blood groups 30 years earlier by Karl Landsteiner faciliated the way to safe transfusions. The first transfusion via blood preserve was performed in 1914 [3, 4]. After this breakthrough, transfusions where disposable for major surgery such as the surgery of the pancreas.

In establishing a method to resect the pancreatic head by mobilizing the duodenum, Theodor Kocher was the forerunner for the huge steps that were made in 1903 [5]. These new techniques helped to perform the first pancreatoduodenectomy by Kausch in a 2-step procedure.

Walter Kausch tried out a two-step pancreatoduodenectomy in 1909. In the first operation, Kausch performed a cholecystojejunostomy and a side-to-side enterostomy. Six weeks later, he resected the head of the pancreas together with the pylorus and the oral two parts of the duodenum. Reconstruction consisted of a gastroenterostomy and anastomosis between the residual parts of the duodenum and the pancreas. The patient survived for 9 months.

Due to the fact that the perioperative care and preoperative diagnosis did not improve as fast as the surgical technique, the definite approach to curative resection was not widespread under surgeons. The high operative risk and late detection of pancreatic cancer led to proceeding more palliative techniques such as a bypass [6].

In 1929, a preoperative diagnosed insulinoma was resected curatively by Roscoe Graham [7]. After hearing about the resection of a neuroendocrine tumor, Whipple saw a reason in a more aggressive approach in patients with carcinoma of the ampulla of Vater and also described a two-step approach. First, he performed a cholecystogastrostomy and posterior loop gastrojejunostomy, followed by a partial duodenectomy, partial pancreatic head resection, and pancreatic stump occlusion weeks later. By observing cholangitis in his patients, he changed his technique into a Roux-en-Y reconstruction of the gastrointestinal passage [8].

He was followed by Verne Hunt and Ridgway Trimble who independently performed a pancreatoduodenectomy in the same year [9, 10]. With findings of the importance of vitamin K and intraoperative blood transfusions, these high-risk methods became safer [6].

Until 1978, the Whipple procedure was standard in pancreatic cancer surgery. Next, Traverso and Longmire introduced the pylorus preserving pancreatoduodenectomy (PPPD) for cancer surgery after it was first described by Kenneth Watson in 1944 to prevent ulcer of the region of the gastrointestinal anastomosis [11].

Both procedures were similar in mortality and morbidity but the PPPD was easier to perform and the operation time was significantly reduced. By preventing the pylorus from resection, the delayed gastric emptying was discussed in several studies with different outcomes [12–14]. In the first years of performing the pancreatoduodenectomy, the mortality rate was approximately 25%. In the 1980's, mortality was reduced in centers with high experience in these procedures to 5% [15].

The first transplantation of the pancreas was successfully performed in 1966 in Minneapolis in a 28-year-old female (the transplant lasted 2 months and was transplanted together with a kidney).

## 2. Establishing Techniques for Chronic Pancreatitis

As a result to the invasive resection and its side effects, the intention lied in modulating resections of the pancreas for benign or premalignant diseases, especially for pancreatitis. In the 16th century, inflammatory processes where described in the pancreas mainly in patients with acute hemorrhagic pancreatitis. Opie discussed the etiology of acute pancreatitis by finding calculi stuck in the ampulla of Vater during autopsies.

Since 1911, it was known that pancreatic drainage and removing calculi from the pancreatic duct relieved pain in patients with chronic pancreatitis. The idea was to treat with less stressful methods. By performing these therapeutic options, the uncertainty of missing a malignant process remains. Therefore, surgeons focused on draining strategies and limited resections.

From 1900 until 1954, there was no clear definition of pancreatitis [16]. Chronic pancreatitis as a disease was first described in 1946 by Comfort [17]. In 1954, first Zollinger et al., then later that year DuVal described the internal retrograde drainage of the pancreas as a pancreaticojejunostomy by performing a distal pancreatectomy with splenectomy, and pancreaticojejunostomy by draining the pancreas with an end to end anastomosis to the jejunum for treatment of chronic pancreatitis [18, 19].

In 1958, Puestow and Gillesby introduced the lateral (longitudinal) opening of the pancreatic duct through the whole organ including the removal of the pancreatic tail and the spleen. The laterolateral pancreaticojejunostomy described by Puestow and Gillesby was an advanced method from the DuVal [18] and Zollinger et al. [20] procedure. They inserted the pancreas in a Roux loop that nearly totally covered it, and the free end of the loop was sewed to the capsule of the pancreas covering the free ending duct [19].

Comparing the radical dimension in resection to the limited advantages in drainage, these approaches seem more or less outdated nowadays. These retrograde drainage procedures aim to reduce the incidence of strictures in the ongoing illness but cause severe trauma. Additionally, there is only a small amount of patients who show single lesions in the pancreatic duct or the pancreatic head for whom these procedures may be indicated [21, 22].

Partington and Rochelle released a paper in 1960, modifying the technique of Puestow and Gillesby in the assumption that the tail of the gland must not necessarily be opened to spare the loss of pancreatic tissue and reduce the loss of endo- and exocrine function of the gland [23–25]. They proposed a longitudinal division of most of the duct staying on the right side of the mesenteric vessels and if possible, opening all sacculations. For reconstruction, a Roux loop is brought retrocolic and opened. The opened jejunum is then attached longitudinally to the pancreas to drain over the whole length. The spleen is preserved in this procedure. In between the years 1958 and 1959, Partington and Rochelle operated 7 patients suffering with pain from chronic pancreatitis and had immediate success documented by pain relief after surgery [19].

In 1972, Beger was the first to perform the duodenum-preserving resection of the head of the pancreas in patients suffering from intractable pain caused by chronic pancreatitis. His procedure spared the gastric resection, the duodenectomy, and the resection of the extrahepatic bile duct while performing a subtotal resection of the pancreatic head and transecting the pancreas above the portal vein without resecting further organs. By interposing a jejunal loop, both parts of the remaining pancreas are drained. A Roux-en-Y reconstruction is performed to restore the gastrointestinal passage. As a result, postoperative morbidity and mortality was lowered. In 1989, Berger published a series of 122 cases with a hospital mortality of 0.8% [26–28]. A study from 2008 shows that the Beger and the Whipple procedure is equally effective in pain control with no differences in survival as well as in endocrine and exocrine parameters [29].

After the Whipple procedure, many patients develop diabetes (28%) and less radical resections are preferred today. The exocrine insufficiency showed the same high percentage (21%) after performing the Whipple resection [30].

Although the early and late mortality of this new technique was very low due to the minor aggressive form of resection, it was only able to remove the inflamed mass in the head of the pancreas. Strictures in the duct remained untouched. Therefore, Frey and Smith modified the Beger procedure in 1985 with a longitudinal incision of the pancreatic duct and a longitudinal pancreaticojejunostomy. Equal to the procedure described by Beger, a small margin of the pancreatic head is left in its place to prevent an injury of organs not involved [31]. Additionally, the hazardous transaction above the portomesenteric axis is avoided. Studies have proven that this procedure can be accomplished with a mortality < 1%; morbidity ranges between 9 and 39% [44, 46], and the pain relief was 90% [47].

The Hamburg procedure was presented in 1998. The advantages of Frey and Beger were combined by undertaking a radical excision of the head and a V-shaped incision of the pancreatic body along with the pancreatic duct. This had the advantage of widening the outlet of the duct and to reach pancreatic side branches [32]. With this modification of the Frey and Beger procedure, the transection of the pancreas above the portal vein and the superior mesenteric vein is avoided and the risk of bleeding reduced. Mortality and morbidity were found to be between 0% and 19.6%, respectively, in a long-term follow-up. 89% of the patients ended up pain free, and the increase of body weight was significantly higher than in the pancreaticoduodenectomy [33].

The Berne procedure (described in 2001 by Büchler) is a modification of the Beger procedure as well. A limited portion of the pancreatic head is resected combined with a pancreaticojejunostomy without a transection above the portal and mesenteric axis.

In contrast to Frey and Hamburg, there is no longitudinal incision of the gland. The cavity of the duct may be widened in case of obstruction, and only one anastomosis is built for reconstruction [34]. Mortality and morbidity for this procedure have shown to be 0% and 20% in a study [54]. As to all duodenum-preserving techniques, the Berne procedure shows equal result regarding postsurgical outcomes. The metabolic complications are reduced as well as survival rates. Furthermore, the Berne procedure is easier to perform than the Beger procedure [31, 35].

Compared to the Beger procedure, no significant difference is shown between both procedures in patient-relevant outcome parameters. The exocrine pancreatic insufficiency was also not significant between the Beger and Berne procedure (83% and 68%). The same results were measured in new onset diabetes between both procedures (Beger: 33%; Berne: 55%).

Additionally, for small duct diseases (in patients without enlarged pancreatic head or dilated duct), there is the Izbicki procedure where a V-shaped excision without further resection is accomplished.

## 3. Present Time

Analyzing the development of surgical techniques treating the pancreas, it can be seen that since the discovery of this organ, there always has been respect approaching it. By step-by-step discovery of different illnesses of the gland without the full knowledge of its function, surgical treatments were not as successful in the beginning. The loss of exocrine and endocrine functions already led to a high mortality not to mention the intraoperative complications.

After discovering insulin and the exocrine juices, surgeons became more successful in the implementation of resections of the pancreas. The substitution of insulin and enzymes as well as the ability of blood transfusions via blood preserve (1922 Banting and Co. were able to save the first patient with diabetes by substituting insulin) improved the outcome dramatically. In pancreatic cancer, the approach to curative treatment includes a radical resection of the part of the pancreas and surrounding structures.

Because of its radicalism, surgeons searched for alternatives treating patients with nonmalignant diseases of the pancreas, such as chronic pancreatitis, pseudocyst, and calculi in the duct. Draining procedures came up very early. After discovering that many of these patients also had strictures in the pancreatic duct, the surgical approach was modified as mentioned above.

## 4. Overview of Surgical Techniques

Nowadays, a wide range of surgical approaches is available for treating patients with chronic pancreatitis (Table 1).

The standard approach for malignant diseases is still early radical surgery. Either the classic Whipple procedure or the pylorus preserving approach is chosen if resectability is given. Although there has been a huge development regarding management of perioperative situations and complications, morbidity still is a central problem in our time. Mortality has decreased to less than 5% in experienced hands [36] but morbidity remains high. Therefore, the evaluations of the right procedure and right time for patients with chronic pancreatitis have to be evaluated carefully.

Detection of tumors and pathological changes in the pancreas have become more sensitive and allow earlier detection through ultrasound and computer tomography and blood chemistry when symptoms occur or by coincidence.

The result of these changes has led to the point that more patients are guided to surgery and indications are provided more generously. Even today, the radical surgical approach is seen as the gold standard for curative therapy.

Focusing on the less invasive techniques for treating chronic diseases or premalignant diseases of the pancreas, a variety of techniques are used which allow a more individual therapeutic concept lowering the effect of pancreatic insufficiency. Studies have shown that a surgical produced drainage of the pancreatic duct showed more effectiveness than endoscopic drainage [23].

Draining procedures such as the Partington-Rochelle procedure or the caudal drainage (Puestow procedure) are rarely chosen as classical treatment due to better outcome of resecting procedures and the missing proof of benign or malignant process. Near total and total pancreatectomy have been put into the background with their extended surgical approach because of high morbidity, mortality, and massive effect on the function of the gland.

Lower morbidity and mortality were found in DPPHR procedures [37]. For patients with an inflammatory mass in the pancreatic head, the pure drainage procedure is not adequate. Until the '70s, a pylorus-preserving pancreaticoduodenectomy (PPPD) was indicated for treatment of the enlarged head of the pancreas.

### 4.1. Minimally Invasive Surgery

The minimally invasive approach is more and more established in surgery. This is a result of its better cosmetic result, less stressfulness for the patient, lower rate of incisional hernias, rapid return of gastrointestinal function, and therefore shorter hospital stay.

The first laparoscopic resection on the pancreas was a pylorus-preserving pancreatic head resection in 1994 in a patient with chronic pancreatitis reported from Michael Gagner. Although feasible, the authors already point out that laparoscopy may not shorten hospital stay or reduce postoperative complications [38].

In a retrospective study between 2008 and 2013, more than 300 patients with ductal adenocarcinoma were either operated on with an open procedure or laparoscopically. The authors sum up that the laparoscopic approach is feasible and shortens hospital stay. The patients that underwent the laparoscopic approach recovered earlier and were able to start with adjuvant therapy sooner [39].

A variety of papers dealing with laparoscopic and robotic approach on pancreatic surgery have been published. Even though widely spread laparoscopy has become the gold standard for cholecystectomy and appendectomy and has become more established in other surgical approaches, laparoscopy of the pancreas is more challenging for the surgeon than any other operation. The learning curve is incomparable to other operations, and therefore, it should only be accomplished by an advanced laparoscopic surgeon in a center with high experience in laparoscopic surgery and pancreatic surgery.

## 5. Gold Standard

Most commonly, a PPPD or DPPHR is selected for the treatment of chronic pancreatitis. The aim of all the duodenum-preserving pancreatic head resections (DPPHR) is to resect the inflammatory mass in the pancreas and spare the unaffected parenchyma of the pancreas to keep as much tissue as possible from the gland, leaving the gastroduodenal passage untouched and the common bile duct continuity unaffected. The mortality after the pancreaticoduodenectomy was shown to be higher than that in the less radical Frey procedure [40].

The Berne and Hamburg procedures were developed by combining the proven advantages of Frey and Beger. Here, the pancreatic head is also resected by protecting the portal vein from injury. In comparison to Frey, the volume of pancreatic head parenchyma is much higher and therefore surely decompressing. In the Hamburg procedure, the body and tail of the gland are additionally opened longitudinally in a V-shaped excision, followed by a side-to-side pancreaticojejunostomy with a branch of the Roux-en-Y draining the duct as Partington and Rochelle described it. Mortality and morbidity for this procedure have proved to be 0% and 20% [54], and the outcomes concerning pain relief is excellent in both procedures [33].

## 6. Future Perspective

Due to the fact that there might not be any further breakthrough in surgical approaches on the pancreas regarding surgical techniques, the future should lie in finding a screening system to differentiate malignancies against enlarged pancreatic heads due to chronic pancreatitis early and preparing patients for surgery.

Minimizing complications such as anastomotic insufficiencies and pancreatic fistulas might lower morbidity significantly in the future and would increase the quality of life of patients with pancreatic diseases.

The Dutch Pancreatitis Study Group is working on a randomized trial evaluating early surgery versus optimal current step-up practice for chronic pancreatitis (ESCAPE) to give a statement for the future treatment of chronic pancreatitis with focus on patients' benefits in terms of pain relief, pancreatic function, and quality of life, compared with the current step-up practice [41].

Today, indications for surgery are very generously seen, leading to an invasive surgical approach that is often followed by a significant reduction of the quality of life. New approaches in perioperative management, focusing on alimentation and mobility, support early recovery and may influence the outcome after pancreatic surgery [42].

An individualized concept is mandatory to evaluate the best surgical approach for the individual patient according to the preoperative diagnostics and intraoperative findings.

## Figures and Tables

**Table 1:** 

Drainage options	(i) Cystojejunostomy
	(ii) Laterolateral pancreaticojejunostomy (Partington-Rochelle procedure)
	(iii) Caudal drainage (Puestow procedure)

Resection	(i) Pancreaticoduodenectomy (PD/Kausch-Whipple procedure) or pylorus preserving pancreaticoduodenectomy (PPPD/Traverso-Longmire-procedure)
	(ii) Duodenum-preserving pancreatic head resection (DPPHR (Beger, Frey, Hamburg, Berne))
	(iii) V-shaped excision
	(iv) Segmental resection
	(v) Distal/total pancreatectomy

Neuroablative procedure	(i) Percutaneus radiofrequency ablation of the splanchnic nerves
	(ii) Thoracoscopic splanchnicectomy
